# Risk Factors for ESBL-Producing *Enterobacteriaceae* in Wastewater of Dairy Farms in East Java, Indonesia

**DOI:** 10.1155/vmi/8706598

**Published:** 2025-09-11

**Authors:** Fidi Nur Aini Eka Puji Dameanti, Sheila Marty Yanestria, Mustofa Helmi Effendi, Wiwiek Tyasningsih, Hani Plumeriastuti, Emmanuel Nnabuike Ugbo, Rahayu Sutrisno, Muhammad Ali Akramsyah Safri

**Affiliations:** ^1^Laboratory of Microbiology and Immunology Veterinary, Faculty of Veterinary Medicine, Universitas Brawijaya, Jl. Puncak Dieng, Kalisongo, Malang Regency 65151, East Java, Indonesia; ^2^Department of Veterinary Public Health, Wijaya Kusuma Surabaya University, Jl. Dukuh Kupang XXV No. 54, Dukuh Kupang, Dukuh Pakis, Surabaya 60225, East Java, Indonesia; ^3^Department of Veterinary Public Health, Faculty of Veterinary Medicine, Universitas Airlangga, Jl. Dr. Ir. H. Soekarno, Kampus C Mulyorejo, Surabaya 60115, East Java, Indonesia; ^4^Department of Veterinary Microbiology, Faculty of Veterinary Medicine, Universitas Airlangga, Jl. Dr. Ir. H. Soekarno, Kampus C Mulyorejo, Surabaya 60115, East Java, Indonesia; ^5^Department of Veterinary Pathology, Faculty of Veterinary Medicine, Universitas Airlangga, Jl. Dr. Ir. H. Soekarno, Kampus C Mulyorejo, Surabaya 60115, East Java, Indonesia; ^6^Department of Applied Microbiology, Faculty of Science, Ebonyi State University, Abakaliki 480211, Nigeria; ^7^Master of Nutrition Science Study Program, Faculty of Health Sciences, Universitas Brawijaya, Jl. Puncak Dieng, Kalisongo, Malang Regency 65151, East Java, Indonesia; ^8^Faculty of Veterinary Medicine, Universitas Brawijaya, Jl. Puncak Dieng, Kalisongo, Malang Regency 65151, East Java, Indonesia

**Keywords:** binomial maps, dairy farms, *Enterobacteriaceae*, ESBL, public health, wastewater

## Abstract

The proliferation of *Enterobacteriaceae* with extended-spectrum beta-lactamase (ESBL), including *Escherichia coli* and *Klebsiella pneumoniae*, poses a significant threat to public health due to their resistance to β-lactam antibiotics. This study focuses on the risk factors of ESBL-producing *Enterobacteriaceae* in the wastewater of dairy farms in East Java, Indonesia, a major milk-producing region. Utilizing an observational cross-sectional design, data were collected from 342 wastewater samples across six regions with high dairy cattle populations in East Java. Risk factors were assessed through questionnaires addressing variables related to farmers and farm management. Univariate analysis by regency/city identified several significant risk factors for the occurrence of ESBL-producing *Enterobacteriaceae* in dairy farm wastewater in East Java, and it differed between regencies/cities. Multivariate analysis of risk factors that significantly correlated with ESBL-producing *Enterobacteriaceae* showed a distance of ≤ 10 m between septic tanks and wells that significantly increased the risk of ESBL-producing *Enterobacteriaceae* occurrence (OR 3.24, 95% CI: 1.07–9.80), as did not using detergent for barn cleaning (OR 2.67, 95% CI: 1.23–5.67). Conversely, the use of water storage tanks and a distance of ≤ 500 m from other dairy farms appeared to reduce the risk. This study provides critical insights for developing strategies to control and prevent antibiotic resistance in the dairy farming sector, such as improved wastewater management, stricter regulations on antibiotic usage, and enhanced farmer education programs. Implementing these strategies is crucial to mitigating the spread of ESBL-producing *Enterobacteriaceae*, thereby improving public and environmental health in East Java.

## 1. Introduction

The production of extended-spectrum beta-lactamase (ESBL) by *Enterobacteriaceae* such as *Escherichia coli* and *Klebsiella pneumoniae* represents a significant global public health challenge. These ESBL-producing bacteria can produce enzymes that break down β-lactam antibiotics, including penicillin and cephalosporins, which are among the most commonly used antibiotics [1]. This resistance diminishes the effectiveness of treatments for bacterial infections and increases the risk of spreading difficult-to-treat diseases [2–4].

One major source of ESBL bacterial spread is agricultural environments, particularly the wastewater generated by dairy farms [5–7]. This wastewater can contain high concentrations of antibiotic-resistant pathogenic bacteria, which can then contaminate surrounding environments, including water sources used by local communities. In East Java, Indonesia, dairy farming is a prominent agricultural activity. The Central Statistics Agency (BPS) noted that East Java is the largest producer of raw milk in Indonesia, which in 2022 produced 108.85 million liters of raw milk. The population of dairy cattle in East Java in 2022 reached 314,385 head, with a high concentration in several regions, including Pasuruan Regency with 100,080 head, Malang Regency with 90,237 head, Tulungagung Regency with 25,836 head, Blitar Regency with 20,209 head, Batu City with 13,053 head, and Kediri Regency with 11,189 head [8]. The high population of dairy cattle needs to be accompanied by increased awareness regarding the use of antibiotics that can be caused by ESBL-producing *Enterobacteriaceae* [9, 10].

The prevalence of ESBL-producing *Enterobacteriaceae* in dairy farm wastewater is a concerning issue in East Java, Indonesia. Several studies have shed light on the prevalence and risk factors associated with ESBL-producing bacteria in dairy farming environments. Gonggrijp et al. [11] conducted a study on a dairy farm to investigate the presence of ESBL- and AmpC-producing *E. coli* in slurry samples, highlighting the importance of bacterial protein production in cattle. Soekoyo et al. [12] focused on the distribution and risk factors of *Enterobacteriaceae* producing ESBL in the gut bacterial flora of dairy cows and individuals living near farming areas in Indonesia, emphasizing the role of farming practices in bacterial spread.

Moreover, Saekhow and Sriphannam [13] explored the prevalence of ESBL-producing *E. coli* strains in dairy farm wastewater, indicating the potential contamination of farm environments with antibiotic-resistant bacteria. Weber et al. [14] identified feeding with waste milk as a significant risk factor for the prevalence of ESBL/AmpC-*E. coli* in preweaned dairy calves, emphasizing the importance of proper waste management in dairy farming practices. Additionally, Waade et al. [15] collected data on antimicrobial use, farm hygiene, and management to identify risks for the occurrence of ESBL-producing bacteria in newborn dairy calves, highlighting the need for comprehensive farm management strategies.

The occurrence of ESBL *E. coli* and *K. pneumoniae* in dairy farm wastewater in East Java, Indonesia, is a critical issue that requires a comprehensive understanding of the risk factors associated with both the farmers and the farming practices. Several studies provide valuable insights into the factors influencing the prevalence of multidrug-resistant bacteria in dairy farms, particularly in East Java, Indonesia. Widodo et al. [16] focused on investigating the potential hazards of multidrug-resistant *E. coli* collected from wastewater on dairy farms in East Java, shedding light on the antibiotic resistance aspect. These findings underscore the importance of addressing antimicrobial resistance in dairy farming practices to mitigate the spread of resistant bacteria in wastewater.

Gonzalez et al. [17] pointed out that direct contact with animal manure and animal slaughter products are potential risk factors for fecal carriage of ESBL-producing *E. coli* and *K. pneumoniae* in both humans and animals. Homeier-Bachmann et al. [18] suggested that improving hygiene management and questioning antibiotic use could help reduce ESBL/AmpC-E. This study aims to identify the risk factors contributing to the high prevalence of these bacteria. These risk factors encompass both aspects related to the farmers and the farming practices.

Farmer-related factors include educational level, income, personal use of antibiotics, sources of drinking water, and the presence of water storage tanks. Farmers' knowledge and health practices significantly impact the management of animal health and farm environment. Farming practice-related factors include the number of dairy cows, the use of antibiotics in livestock, sanitation practices, the use and maintenance of farming tools, and the management of manure and wastewater. Farms with high livestock densities and poor sanitation practices are more likely to have higher risks of spreading resistant bacteria. The results of this research are expected to provide a clear picture of the risk factors influencing the spread of ESBL bacteria in the dairy farm environment. Consequently, this study can offer a scientific basis for controlling and preventing the spread of antibiotic resistance in the agricultural sector and improve environmental and public health in East Java.

## 2. Materials and Methods

### 2.1. Study Design

This research is a continuation of the research published in February and November 2023 [19, 20], which uses an observational study with cross-sectional data collected in June 2022. A cross-sectional study design was chosen as it allows for simultaneous measurement of risk factors and the presence of ESBL-producing *Enterobacteriaceae* at a single point in time. This approach is suitable for identifying potential risk factors associated with ESBL occurrence without requiring long-term follow-ups, making it practical and efficient for field-based epidemiological studies.

The sample size was determined using the disease prevalence formula by Martin et al. [21], as follows:

(1) $$\begin{array}{c} N=4\text{pq}:\alpha^{2}. \end{array}$$

where prevalence (p): 16% (based on [22], a study on ESBL-producing *E. coli* in dairy farm wastewater), confidence level: 95%, and margin of error (*α*): 0.1.

Based on this calculation, the minimum required sample size per region was 54, leading to a total of 324 samples. However, to increase representativeness and statistical power, we collected 342 samples. While this sample size may not capture all potential risk factors, it is sufficient for initial epidemiological assessment [21]. Future studies should consider larger sample sizes to validate these findings.

### 2.2. Participation Criteria

A nonprobability sampling technique, specifically purposive sampling, was employed in this study. This method was chosen because the study aimed to target dairy farms with specific characteristics related to antibiotic use and farm management practices. The inclusion criteria were as follows:

1. Dairy farms located in the six districts/cities with the highest dairy cattle populations in East Java (Pasuruan District, Malang District, Tulungagung District, Blitar District, Batu City, and Kediri District).
2. Farms with a recorded history of antibiotic use, identified through surveys and consultation with veterinary officers and cooperative units (KUD), but only farmers that agreed to participate in this study were included. The dairy farmer signed an informed consent form before being interviewed using a questionnaire.

By using purposive sampling, we ensured that the selected farms were relevant for assessing the relationship between risk factors and ESBL-producing *Enterobacteriaceae* [22]. While this approach may limit generalizability, it is effective for exploratory epidemiological studies where targeted sampling is necessary.

### 2.3. Ethical Approval and Informed Consent

Ethical approval for this study was obtained from the Health Research Ethics Committee of Universitas Brawijaya, Indonesia, under approval number **196-KEP-UB-2023**. All procedures involving human participants were conducted in accordance with applicable ethical standards. Prior to data collection, **written informed consent** was obtained from all participants. Each participant was fully informed about the purpose, procedures, and voluntary nature of the study and provided their consent by signing a consent form.

### 2.4. Location of the Farms

**Figure 1:**
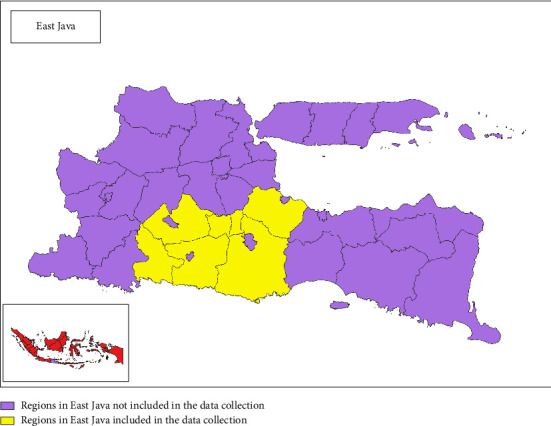
Location of farms that participated in the study.

To illustrate the location of farms that participated in the study, a map was created using QGIS. The farms were mainly located in East Java, Indonesia (Figure 1).

### 2.5. ESBL-Producing *Enterobacteriaceae*

ESBL-producing *Enterobacteriaceae* were obtained from 2 types of bacteria, namely, *K. pneumoniae* and *E. coli*. *K. pneumoniae* produces ESBL, according to the previous study by Dameanti et al. [20], and for *E. coli* producing ESBL, it is based on a previous publication from February 2023 [19], followed by testing using polymerase chain reaction (PCR) [20].

### 2.6. Questionnaire and Data Collection

The questionnaire collected information regarding potential risk for the occurrence of *Enterobacter* ESBL within the farm. The farmers were interviewed individually by the researcher. The researcher reads the questions, and the farmers answer them one by one. The questions in the questionnaire were divided into two groups, which had 40 questions (Table 1).

Risk factors for the occurrence of ESBL-producing *Enterobacteriaceae* are divided into two groups of variables: farmers and farms. The farmer variable group consists of risk factors originating from the farmers themselves, either directly related to the individual farmers or from their residences. The second group of risk factors is farm management. This group includes risk variables from the livestock side, encompassing the animals themselves, management practices, and the equipment used.

### 2.7. Data Analysis

All statistical analyses were carried out using IBM SPSS Statistics Version 22.0. The occurrence of ESBL-producing *Enterobacteriaceae* was categorized by 2 groups (binomial). Logistic regression was applied to analyze the influence of the binomial parameters on the occurrence of ESBL-producing *Enterobacteriaceae*. We classified the occurrence of ESBL-producing *Enterobacteriaceae* and the risk factor variable as positive or negative. The risk factor analysis was conducted twice. The first analysis of risk factors was based on regencies/cities, and the second was based on provinces or the entire sample. The risk factors were analyzed using univariate and multivariate analyses. The univariate analysis was performed using Pearson's chi-square test, and the relationship between risk factors and the occurrence of ESBL-producing *Enterobacteriaceae* was analyzed using the odds ratio (OR) with a 95% confidence interval (CI). Variables with a *p*-value < 0.05 were selected as candidates for multivariate analysis. The multivariate analysis was conducted to determine the correlation between risk factors, where a *p*-value < 0.05 indicates statistical significance [23]. The multivariate analysis is just used in provinces or the entire sample test.

## 3. Result and Discussion

**Figure 2:**
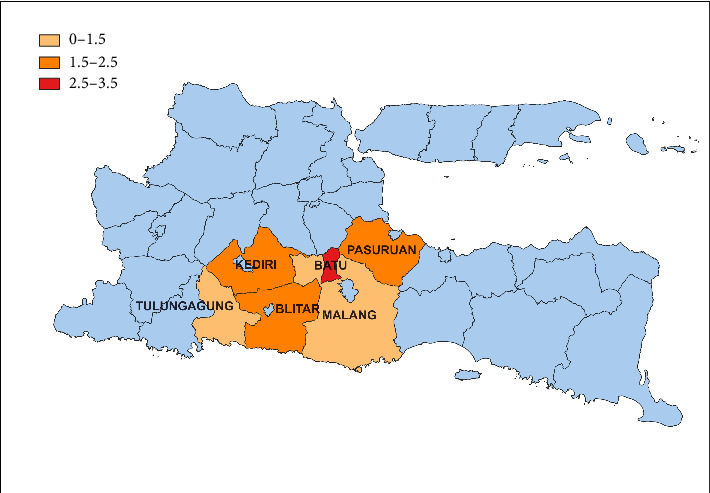
Percentage of ESBL-producing *Enterobacteriaceae* in dairy farm wastewater in East Java.

The data of occurrence of ESBL-producing *Enterobacteriaceae* are presented in Table 2 and Figure 2. In this study, the occurrence of ESBL-producing *Enterobacteriaceae* was 12.34%, while the occurrence of non-ESBL *Enterobacteriaceae* was 87.76%. The highest occurrence of ESBL-producing *Enterobacteriaceae* was in Batu City, reaching 3.50%, and the lowest occurrence was in Malang Regency, at 1.05%.

The result of the risk factor ESBL-producing *Enterobacteriaceae* is shown in Table 3. Univariate analysis by regency/city identified several significant risk factors for the occurrence of ESBL-producing *Enterobacteriaceae* in dairy farm wastewater in East Java, and it differed between regencies/cities (Table 4). The critical factors in Kediri Regency were handwashing before milking and using soap. The significant factor in Malang Regency was just the farmer's history of antibiotic use. Blitar Regency's significant factors included the presence of dispersed solid waste in the milking area and wastewater disposal pathways. Pasuruan Regency has the type of water used for livestock maintenance, and the drinking water source for cows was significant. Tulungagung Regency had multiple significant factors: the barn's distance from dairy farms, the type of bathroom wastewater disposal system, the drinking water source for the farmer, the water source for nonconsumption activities, and the proximity to the nearest healthcare facility. Batu City identified the frequency of barn cleaning, washing the cows/udder, the disposal of solid waste to other farms, and the use of clean equipment in livestock handling as critical factors.

The univariate analysis based on city/regency reveals discrepancies compared to the analysis conducted for the entire sample or at the provincial level. In this result, variables from the farmers' group exhibit *ρ* values ranging from 0.03 to 0.08. Notably, among these variables, the significant factor influencing ESBL occurrence from the farmers' group is the distance between the septic tank and the well (*ρ* value < 0.03). Variables from the farms group showcase *ρ* values from 0.01 to 0.98, with significant variables registering *ρ* values of 0.01, 0.03, and 0.04. These variables include the distance to the nearest dairy farm, the utilization of water storage tanks, and the application of detergent for barn cleaning. Subsequently, these four variables underwent multivariate analysis to unveil correlations among them, with the resulting outcomes elucidated in Table 5.

Table 5 showed the result of multivariate analysis. It indicates that a distance of ≤ 10 m between the septic tank and the well poses a threefold risk (OR 3.24, 95% CI: 1.07–9.80, *p* = 0.04). This finding underscores the role of groundwater contamination in bacterial spread, as poorly managed septic systems can introduce antibiotic-resistant bacteria into the water supply [24]. Additionally, farms that did not use detergent for barn cleaning had an increased risk (OR 2.67, 95% CI: 1.23–5.67), highlighting the importance of proper hygiene in reducing bacterial persistence [25]. Conversely, the presence of water storage tanks and proximity to other dairy farms (< 500 m) appeared to reduce ESBL risk, possibly due to improved water management and biosecurity practices.

## 4. Discussion

The occurrence of ESBL-producing *Enterobacteriaceae* in this study is lower than that found in studies by Maulana et al. [22] and Santman-Berends et al. [26], where the ESBL occurrence from dairy cow farm feces in Yogyakarta reached 25% and from organic dairy farm slurry in the Netherlands reached 13%. Overall, the ESBL occurrence in each area studied is still higher than in Heuvelink et al. [27], where individual analysis showed that fecal samples from dairy cows in the Netherlands had an ESBL prevalence of 0.8%. The high occurrence of ESBL should be a public health concern, as these bacteria can be zoonotic and transmitted to humans through various routes. Contaminated environments with ESBL-producing bacteria can potentially spread these bacteria to humans [5].

The risk factors of occurrence of ESBL-producing *Enterobacteriaceae* in dairy farm wastewater vary significantly across different regions in East Java, including Kediri, Malang, Blitar, Pasuruan, Tulungagung, and Batu. This variation is driven by a number of interrelated factors. First, variations in farm management practices, particularly with respect to the protocols for antibiotic use, hygiene levels, and waste systems, are an important factor. The occurrence of ESBL is lower in regions with stricter antibiotic regulations, better adherence to veterinary guidelines, and improved waste treatment facilities. Second, the implementation of effective farm management practices is determined by demographic and economic factors, like education and income levels of farmers. Higher farmer education and income correlate with better management practices and lower ESBL prevalence in these areas. Thus, the regional diversity of ESBL-producing *Enterobacteriaceae* occurrence in East Java's dairy farm wastewater is a complex problem that requires holistic and targeted intervention to tackle the various influencing factors involved.

On the first variable, the group of risk factors of ESBL-producing *Enterobacteriaceae* is the farmers. In this variable group, the distance between septic tanks and wells, which is ≤ 10 m, had statistical significance, with an ESBL occurrence rate of 88.57% (31 occurrences). Septic tanks within 10 m pose a threefold risk for ESBL-producing *Enterobacteriaceae*. Conventional septic tanks collect wastewater from toilets and allow feces to settle and seep slowly into the soil, causing microbiological contamination of groundwater. Groundwater contamination is indicated by the presence of *E. coli* from septic tank waste, where close proximity to groundwater prevents proper filtration, affecting surrounding water quality [28]. Studies show a significant relationship between septic tank distance and *E. coli* content in well water, which is at risk of pathogenic contamination [24, 29]. *E. coli* in spring water indicates the presence of pathogenic bacteria [30]. Contaminated environments are key factors in gene mutations. Reference [31] showed that the environment is crucial in the transmission and emergence of resistant bacteria. Environmental stress can also cause *E. coli* resistance gene development [32].

The second variable group of risk factors of ESBL-producing *Enterobacteriaceae* is from the farms. The risk factors that are statistically significant are the distance to the nearest dairy farm, the utilization of water storage tanks, and the application of detergent for barn cleaning. The distance to other dairy farms within 500 m has a statistically significant impact (*p*-value of 0.01). Close farm locations to others can lead to waste (feces or urine) entering other farms via water flow, especially during the rainy season. Dairy cows can spread antibiotic-resistant bacteria through feces [33]. These bacteria can migrate around the farm, contaminating even distant environments [34]. Additionally, pets from other barns can spread ESBL-producing *Enterobacteriaceae* between farms since pets are often free-ranging. Studies by Benavides et al. [35] found ESBL genes in chickens, cattle, dogs, and rodents. ESBL-producing *E. coli* prevalence was significantly high in dogs (24% [CI: 16%–35%]; 20 of 82).

Water-related risk factors play a crucial role in the occurrence of ESBL-producing *Enterobacteriaceae* in dairy farm wastewater in East Java. As it has been demonstrated earlier, the variable associated with water tank storage units is again statistically significant, and it is also a risk factor for the presence of ESBL in *Enterobacteriaceae*. Further supporting this, research by Gay et al. [36] noted a 0.38 times increase in the occurrence of ESBL *E. coli* due to the presence of water storage tanks in dairy settings. Looking to the future, it is suspected that this contamination is caused by sediment accumulation in the tanks. van der Wielen and Lut [37] and Liu et al. [38] showed that sediments from water distribution systems had organic nitrogen and high amounts of bacteria and invertebrates, which nourished and sheltered bacteria. Sediment traps prevent bacteria from being exposed to high storage temperatures, fecal material contamination, and degradation of water quality [38]. The survey clientele of Tsholo et al. [39] included raw and treated drinking water from facilities for the production of drinking water in the North West Province of South Africa and appeared to contain antibiotic resistance genes and antibiotics, especially in rare and treated water, which shows that antibiotic resistance is related to one of the environmental sources of drinking water.

A further important risk factor is the insufficiency of detergent use and improper cleaning of barn areas. Use of detergent, which was related to barn cleaning, and improper cleaning techniques were highly significant (*p*-values 0.04 and 0.06, respectively). Detergents are chemicals that can inhibit the growth of microorganisms, even pathogenic bacteria, on surfaces and are critical in the prevention of communicable diseases [25]. Cleaning with detergents is important in curtailing the spread of antibiotic-resistant bacteria [40]. However, when overused, detergents cause resistance of microorganisms. Hamdi and Sami [40] have found a direct relationship between the period of exposure and the detergent sensitivity of the bacterial strains tested. Out of 15 types of detergents tested, all of them inhibited some degree of growth of bacteria, but the bactericidal effect was found to be weak to strong in all the tested cases. These findings corroborated with the present study in supporting the fact that the use of compounds that contain detergent to wash all surfaces of the barn reduces the chances of occurrence of ESBL. Also [41], in their study, reported that floors of dairy barns, if cleaned correctly, are devoid of ESBL.

While many risk factors did not significantly influence the occurrence of ESBL, they still warrant attention. Education: About 60% of ESBL occurrences occurred in farmers who did not meet mandatory education requirements (until senior high school). Although education level affects knowledge of farm management and antibiotic use, statistical results were not significant, aligning with findings from Soekoyo et al. [12] and Suherman et al. [42]. Income: Approximately 51.43% of ESBL occurrences were among farmers with incomes below the East Java minimum wage (Rp. 1,891,567.12). Low incomes limit access to resources, impeding proper farm management and increasing the risk of antibiotic resistance [10]. Antibiotic use history: Farmers' antibiotic consumption accounted for 22.86% of occurrences, as direct contact without proper hygiene facilitates resistant bacteria transfer to livestock [43]. Bathroom drainage and water Source: Open drainage systems caused 60% of occurrences, as they allow antibiotics from human waste to contaminate the environment [44]. Non-PDAM water sources, used by 60% of farms, are more susceptible to contamination from resistant bacteria, with wild animals and polluted environments serving as additional reservoirs [45]. Accessibility and livestock factors: Farms located over 500 m from healthcare facilities had 80% of occurrences, highlighting limited healthcare access as a risk factor for unsupervised antibiotic use [46]. Farms with over five cows or only one barn also showed higher ESBL rates due to crowding, waste accumulation, and poor sanitation [47]. Hygiene practices: Shared equipment and poor hygiene, such as failure to wash hands before milking (88.57% of occurrences), contributed to bacterial spread [48]. Regular barn cleaning did not fully mitigate risks, likely due to contaminated water sources and substandard cleaning practices.

While several of these factors did not reach statistical significance, this may be attributed to sample size limitations. A larger sample size could improve statistical power, potentially revealing significant associations that were not detected in this study. Additionally, stratifying samples based on farm management practices and antibiotic usage patterns may offer deeper insights into the transmission dynamics of ESBL-producing Enterobacteriaceae. Future research should consider expanding sample sizes and incorporating longitudinal assessments to better evaluate the long-term impact of these risk factors on antimicrobial resistance trends in dairy farms.

In conclusion, this study highlights key risk factors contributing to the presence of ESBL-producing Enterobacteriaceae in dairy farm wastewater. However, some risk factors did not show statistical significance, which may be due to the sample size limitations. Future studies with larger sample sizes and a more detailed assessment of farm-level antibiotic use and sanitation practices are needed to validate these findings. Addressing these factors through improved farm management, stricter antibiotic regulations, and hygiene practices can help mitigate the spread of antibiotic resistance in dairy farms.

## Figures and Tables

**Table 1:** Risk factor variable category.

No	Variables for farmers	Variables for farms
1	Education level	Number of dairy cows
2	Income	Number of barns
3	History of antibiotic use	Nearest water source
4	Bathroom wastewater disposal	Distance to nearest water source
5	Drinking water source	Distance of the barn to the river
6	Water source for nonconsumption needs	Other animals in the barn
7	Distance from septic tank to well	Distance to the nearest dairy farm
8	Distance from nearest healthcare facility	Distance to the nearest nondairy farm
9		Milking by farmers from other farms
10		Sharing milking equipment between livestock
11		Handwashing before milking
12		Hand washing with soap
13		Barn cleaning
14		Livestock cleaning
15		Water usage for barn cleaning
16		Detergent use for barn cleaning
17		Surface cleaning
18		Type of water used for livestock maintenance
19		Use of water storage tanks
20		Drinking water source for livestock
21		Presence of rodents
22		Quarantine of livestock before entering the barn
23		Separation of sick livestock from healthy ones
24		Manure piles in the milking area
25		Spread of solid waste in the milking area
26		Disposal of solid waste to other farms
27		Wastewater disposal pathways
28		Wastewater puddles around the barn
29		Presence of wastewater treatment
30		History of antibiotic use in livestock
31		Type of feed
32		Hand milking system

**Table 2:** Occurrence of ESBL-producing *Enterobacteriaceae* in dairy farm wastewater.

Cities/regencies	ESBL		Non-ESBL		Total	
	*n*	%	*n*	%	*n*	%
Kediri	5	1.75	45	15.73	50	17.48
Blitar	6	2.10	33	11.54	39	13.64
Malang	3	1.05	58	20.28	61	21.33
Batu City	10	3.50	37	12.94	47	16.43
Pasuruan	7	2.45	47	16.43	54	18.88
Tulungagung	4	1.40	31	10.84	35	12.24

East Java	35	12.24	251	87.76	286	100

**Table 3:** Risk factor data of occurrence of ESBL-producing *Enterobacteriaceae* in dairy farm wastewater.

Risk factors	Kediri		Blitar		Malang		Batu City		Pasuruan		Tulungagung		Total	
	ESBL (*n* = 5)	No-ESBL (*n* = 45)	ESBL (*n* = 6)	No-ESBL (*n* = 33)	ESBL (*n* = 3)	No-ESBL (*n* = 58)	ESBL (*n* = 10)	No-ESBL (*n* = 37)	ESBL (*n* = 7)	No-ESBL (*n* = 47)	ESBL (*n* = 4)	No-ESBL (*n* = 31)	ESBL (*n* = 35)	No-ESBL (*n* = 251)
*Farmers*														
Education level														
Nonmandatory education level	0	13	6	19	2	48	5	19	6	28	2	18	21	145
Mandatory education level	5	32	0	14	1	10	5	18	1	19	2	13	14	106
Income														
≤ Minimum wage	5	36	1	19	2	43	5	21	2	24	3	18	18	161
> Minimum wage	0	9	5	14	1	15	5	16	5	23	1	13	17	90
History of antibiotic use														
Yes	0	0	0	4	1	2	1	5	3	18	3	17	8	46
No	5	45	6	29	2	56	9	32	4	29	1	14	27	205
Bathroom wastewater disposal														
Open	0	0	6	29	1	38	7	27	6	46	1	24	21	164
Closed	5	45	0	4	2	20	3	10	1	1	3	7	14	87
Drinking water source														
Non-PDAM	5	45	0	4	2	20	3	10	1	1	3	7	14	87
PDAM	0	0	6	29	1	38	7	27	6	46	1	24	21	164
Water source for nonconsumption needs														
Non-PDAM	0	0	6	30	1	31	7	27	6	45	1	24	21	157
PDAM	5	45	0	3	2	27	3	10	1	2	3	7	14	94
Distance from septic tank to well														
≤ 10 m	5	45	5	18	3	23	7	31	7	31	4	30	31	178
> 10 m	0	0	1	15	0	35	3	6	0	16	0	1	4	73
Distance from nearest healthcare facility														
≤ 500 m	0	0	0	0	1	15	1	5	1	5	1	0	4	25
> 500 m	5	45	6	33	2	43	9	32	6	42	3	31	31	226

*Farms*														
Number of dairy cows														
< 5 cows	3	24	0	7	1	13	4	15	2	17	2	9	12	85
≥ 5 cows	2	21	6	26	2	45	6	22	5	30	2	22	23	166
Number of barns														
1 barn	4	40	6	21	1	51	9	34	7	46	3	24	30	216
> 1 barn	1	5	0	12	2	7	1	3	0	1	1	7	5	35
Nearest water source														
Stagnant water	4	42	6	31	3	48	9	36	7	47	4	30	33	234
Flowing water	1	3	0	2	0	10	1	1	0	0	0	1	2	17
Distance to nearest water source														
≤ 100 m	1	6	1	2	1	32	2	2	1	9	0	0	6	51
> 100 m	4	39	5	31	2	26	8	35	6	38	4	31	29	200
Distance of the barn to the river														
≤ 500 m	0	0	0	0	0	4	2	4	4	20	0	5	6	33
> 500 m	5	45	6	33	3	54	8	33	3	27	4	26	29	218
Other animals in the barn														
Yes	5	43	6	32	3	52	9	31	7	47	4	31	34	236
No	0	2	0	1	0	6	1	6	0	0	0	0	1	15
Distance to the nearest dairy farm														
≤ 500 m	2	24	2	21	2	47	8	33	4	40	2	28	20	193
> 500 m	3	21	4	12	1	11	2	4	3	7	2	3	15	58
Distance to the nearest nondairy farm														
≤ 500 m	0	6	2	13	1	39	10	36	7	42	3	26	23	162
> 500 m	5	39	4	20	2	19	0	1	0	5	1	5	12	89
Milking by farmers from other farms														
Yes	1	3	0	0	0	4	0	2	1	9	1	2	3	20
No	4	42	6	33	3	54	10	35	6	38	3	29	32	231
Sharing milking equipment between livestock														
Yes	1	17	1	17	0	11	1	7	2	14	4	31	9	97
No	4	28	5	16	3	47	9	30	5	33	0	0	26	154
Hand washing before milking														
No	2	43	6	33	3	45	9	33	7	47	4	31	31	232
Yes	3	2	0	0	0	14	1	4	0	0	0	0	4	20
Handwashing with soap														
No	3	9	0	0	0	23	1	4	1	2	0	0	5	38
Yes	2	36	6	33	3	35	9	33	6	45	4	31	30	213
Barn cleaning														
Rarely	0	1	0	0	0	10	3	0	0	0	0	0	3	11
Always	5	44	6	33	3	48	7	37	7	47	4	31	32	240
Livestock cleaning														
Rarely	0	1	0	2	0	4	3	0	1	7	0	0	4	14
Always	5	44	6	31	3	54	7	37	6	40	4	31	31	237
Water usage for barn cleaning														
Non-PDAM	4	35	5	31	2	25	10	32	6	44	4	28	31	195
PDAM	1	10	1	2	1	33	0	5	1	3	0	3	4	56
Detergent use for barn cleaning														
No	1	38	4	16	3	34	2	19	3	22	1	17	14	146
Yes	4	7	2	17	0	24	8	18	4	25	3	14	21	105
Surface cleaning														
Partial	2	21	0	11	1	20	0	4	0	0	0	0	3	56
Complete	3	24	6	22	2	38	10	33	7	47	4	31	32	195
Type of water used for livestock maintenance														
Non-PDAM	4	36	6	31	1	28	8	30	6	47	4	29	29	201
PDAM	1	9	0	2	2	30	2	7	1	0	0	2	6	50
Use of water storage tanks														
Yes	1	21	2	16	0	32	3	14	3	28	2	17	11	128
No	4	24	4	17	3	26	7	23	4	19	2	14	24	123
Drinking water source for livestock														
Non-PDAM	4	36	6	31	1	23	8	31	6	47	4	29	29	197
PDAM	1	9	0	2	2	35	2	6	1	0	0	2	6	54
Presence of rodents														
Yes	3	34	5	26	2	48	6	31	7	34	4	22	27	195
No	2	11	1	7	1	10	4	6	0	13	0	9	8	56
Quarantine of livestock before entering the barn														
No	5	40	6	33	2	48	8	35	7	47	4	30	32	233
Yes	0	5	0	0	1	10	2	2	0	0	0	1	3	18
Separation of sick livestock from healthy ones														
No	5	36	1	8	0	32	6	23	7	40	1	7	20	146
Yes	0	9	5	25	3	26	4	14	0	7	3	24	15	105
Manure piles in the milking area														
Yes	1	10	5	25	1	37	8	29	2	16	4	26	21	143
No	4	35	1	8	2	21	2	8	5	31	0	5	14	108
Spread of solid waste in the milking area														
Yes	1	10	4	31	0	19	8	29	4	34	2	22	19	145
No	4	35	2	2	3	39	2	8	3	13	2	9	16	106
Disposal of solid waste to other farms														
Yes	0	1	0	0	0	8	3	2	0	1	0	0	3	12
No	5	44	6	33	3	50	7	35	7	46	4	31	32	239
Wastewater disposal pathways														
No	0	5	1	2	1	8	0	0	0	3	0	0	2	18
Yes	5	40	5	31	2	50	10	37	7	44	4	31	33	233
Wastewater puddles around the barn														
Yes	1	13	6	21	0	21	8	24	2	18	2	27	19	124
No	4	32	0	12	3	37	2	13	5	29	2	4	16	127
Presence of wastewater treatment														
Yes	5	26	6	31	2	43	7	20	5	32	2	12	27	164
No	0	19	0	2	1	15	3	17	2	15	2	19	8	87
History of antibiotic use in livestock														
Yes	5	38	6	26	3	58	10	34	5	43	3	23	31	222
No	0	7	0	7	0	0	0	3	2	4	1	8	3	29
Type of feed														
Full forage	1	7	0	0	0	5	0	3	1	11	0	7	2	33
Additional feed	4	38	6	33	3	53	10	34	6	36	4	24	33	218
Hand milking system														
Hand milking	5	39	6	25	3	53	7	31	5	39	3	27	29	214
Machine milking	0	6	0	8	0	5	3	6	2	8	1	4	6	37

**Table 4:** Univariate analysis of risk factor of occurrence of ESBL-producing *Enterobacteriaceae* in dairy farm wastewater.

Risk factors	Cities/regencies						Total		
	Kediri	Blitar	Malang	Batu City	Pasuruan	Tulungagung			
	*ρ*	*ρ*	*ρ*	*ρ*	*ρ*	*ρ*	Crude OR	95% CI	*ρ*
Farmers									
Education level	0.16	0.19	0.50	0.94	0.18	0.76	0.91	0.44–1.88	0.80
Income	0.27	0.18	0.44	0.70	0.27	0.52	1.69	0.83–3.44	0.15
History of antibiotic used	—	0.40	0.01^∗^	0.77	0.82	0.44	0.78	0.32–1.77	0.52
Bathroom wastewater disposal	—	0.40	0.06	0.85	0.11	0.03^∗^	1.26	0.61–2.59	0.54
Drinking water source	—	0.40	0.06	0.85	0.11	0.03^∗^	1.26	0.61–2.59	0.54
Water source for nonconsumption needs	—	0.40	0.13	0.85	0.28	0.03^∗^	0.90	0.44–1.85	0.77
Distance from septic tank to well	—	0.11	0.95	0.33	0.07	0.72	0.32	0.11–0.92	0.03^∗^
Distance from nearest healthcare facility	—	—	0.44	0.77	0.78	0.01^∗^	0.86	0.28–2.63	0.79
Farms									
Number of dairy cows	0.78	0.78	0.43	0.98	0.69	0.40	0.98	0.47–2.07	0.96
Number of barns	0.56	0.30	0.15	0.85	0.70	0.91	1.03	0.37–2.83	0.96
Nearest water source	0.30	0.64	0.50	0.31	—	0.72	1.20	0.27–5.43	0.81
Distance to nearest water source	0.68	0.47	0.91	0.14	0.76	—	1.23	0.49–3.13	0.66
Distance of the barn to the river	—	—	0.70	0.44	0.50	0.39	0.73	0.28–1.90	0.52
Other animals in the barn	0.63	0.64	0.64	—	—	—	0.46	0.06–2.62	0.45
Distance to the nearest dairy farm	0.57	0.07	0.48	0.44	0.08	0.03^∗^	2.5	1.20–5.18	0.01^∗^
Distance to the nearest nondairy farm	0.38	0.55	0.64	0.60	0.37	0.66	0.95	0.45–1.99	0.89
Milking by farmers from other farms	0.30	—	0.70	0.45	0.76	0.21	0.92	0.26–3.28	0.90
Sharing milking equipment between livestock	0.43	0.06	0.50	0.51	0.95	—	1.82	0.82–4.05	0.14
Handwashing before milking	0.00^∗^	—	0.43	0.94	—	—	0.67	0.22–2.09	0.49
Handwashing with soap	0.04^∗^	—	0.26	0.94	0.28	—	0.93	0.34–2.56	0.90
Barn cleaning	0.74	—	0.52	0.00^∗^	—	—	0.50	0.13–1.85	0.28
Livestock cleaning	0.74	0.50	0.70	0.00^∗^	0.97	—	0.46	0.14–1.48	0.18
Water usage for barn cleaning	0.91	0.50	0.22	0.22	0.46	0.52	2.23	0.75–6.57	0.14
Detergent use for barn cleaning	0.80	0.62	0.64	0.08	0.85	0.26	0.48	0.23–0.97	0.04^∗^
Surface cleaning	0.78	0.06	0.95	0.23	—	—	0.33	0.10–1.11	0.06
Type of water used for livestock maintenance	1.00	0.50	0.17	0.94	0.01^∗^	0.60	1.20	0.47–3.05	0.70
Use of water storage tanks	0.25	0.30	0.13	0.65	0.40	0.86	2.27	1.07–4.83	0.03^∗^
Drinking water source for livestock	1.00	0.50	0.25	0.78	0.01^∗^	0.60	1.33	0.52–3.36	0.55
Presence of rodents	0.45	0.56	0.50	0.10	0.11	0.21	1.03	0.44–2.40	0.94
Quarantine of livestock before entering the barn	0.43	—	0.23	0.14	—	0.72	0.82	0.23–2.96	0.77
Separation of sick livestock from healthy ones	0.27	0.54	0.13	0.90	0.27	0.92	0.96	0.47–0.196	0.91
Manure piles in the milking area	0.91	0.70	0.72	0.91	0.78	0.39	0.88	0.43–1.82	0.73
Spread of solid waste in the milking area	0.91	0.00^∗^	0.33	0.91	0.41	0.40	1.15	0.57–2.35	0.70
Disposal of solid waste to other farms	0.74	—	0.58	0.03^∗^	0.70	—	0.54	0.14–2.00	0.35
Wastewater disposal pathways	0.43	0.02^∗^	0.55	—	0.49	—	0.79	0.17–3.45	0.75
Wastewater puddles around the barn	0.43	0.30	0.30	0.36	0.62	0.06	0.82	0.40–1.67	0.59
Presence of wastewater treatment	0.07	0.50	0.44	0.37	0.86	0.66	1.79	0.78–4.12	0.17
History of antibiotic use in livestock	0.34	0.78	0.85	0.35	0.12	0.97	0.99	0.33–3.00	0.98
Type of feed	0.80	—	0.67	0.35	0.59	0.29	2.50	0.57–10.90	0.21
Hand milking system	0.38	0.17	0.64	0.33	0.46	0.52	1.20	0.47–3.08	0.71

Abbreviations: CI = confidence interval; crude OR = crude odds ratio.

∗*ρ* < 0.05.

**Table 5:** Multivariate analysis of risk factors of occurrence of ESBL-producing *Enterobacteriaceae* in dairy farm wastewater.

Variable	Adjusted OR	95% CI	*ρ*
Farmer			
Distance from septic tank to well ≤ 10 m	3.24^∗^	1.07–9.80	0.04
Farms			
Distance to the nearest dairy farm ≤ 500 m	0.50	0.23–1.08	0.07
Detergent use for barn cleaning	2.67^∗^	1.23–5.67	0.01
Use of water storage tanks	0.43^∗^	0.19–0.94	0.03

Abbreviations: adjusted OR = adjusted odds ratio; CI = confidence interval.

∗*ρ* < 0.05.

## Data Availability

The datasets used and analyzed in this study are available from the corresponding author upon reasonable request.
